# Pipeline Monitoring Using Highly Sensitive Vibration Sensor Based on Fiber Ring Cavity Laser

**DOI:** 10.3390/s21062078

**Published:** 2021-03-16

**Authors:** Nageswara Lalam, Ping Lu, Abhishek Venketeswaran, Michael P. Buric

**Affiliations:** 1 National Energy Technology Laboratory, 626 Cochrans Mill Road, Pittsburgh, PA 15236, USA; Nageswara.Lalam@netl.doe.gov (N.L.); abhishek.venketeswaran@netl.doe.gov (A.V.); 2 Leidos, 626 Cochrans Mill Road, Pittsburgh, PA 15236, USA; 3 National Energy Technology Laboratory, 3610 Collins Ferry Road, Morgantown, WV 26505, USA; Michael.Buric@netl.doe.gov

**Keywords:** fiber ring laser, pipeline monitoring, vibration sensing

## Abstract

A vibration fiber sensor based on a fiber ring cavity laser and an interferometer based single-mode-multimode-single-mode (SMS) fiber structure is proposed and experimentally demonstrated. The SMS fiber sensor is positioned within the laser cavity, where the ring laser lasing wavelength can be swept to an optimized wavelength using a simple fiber loop design. To obtain a better signal-to-noise ratio, the ring laser lasing wavelength is tuned to the maximum gain region biasing point of the SMS transmission spectrum. A wide range of vibration frequencies from 10 Hz to 400 kHz are experimentally demonstrated. In addition, the proposed highly sensitive vibration sensor system was deployed in a field-test scenario for pipeline acoustic emission monitoring. An SMS fiber sensor is mounted on an 18” diameter pipeline, and vibrations were induced at different locations using a piezoelectric transducer. The proposed method was shown to be capable of real-time pipeline vibration monitoring.

## 1. Introduction

An increase in pipeline vibrations is often indicative of hazardous conditions, such as (i) gas leaks, (ii) loose pipeline connections, and (iii) corrosion induced structural damage. Such hazardous events often trigger vibration signatures that propagate over a localized spatial extent and can lead to down-time for large segments of the pipeline network. Gas leaks are often accompanied by hissing sounds and have pronounced low-frequency acoustic signatures [1], while other pipeline wall defects (cracks, notches) are known to scatter ultrasonic guided waves that travel along the pipe [2]. Continuous distributed monitoring of pipeline vibrations is necessary for their predictive maintenance, to assess their real-time structural health, and to enable early fault detection to ensure safe operational conditions. Ultrasonic vibration detection using fiber optic sensors gained attention due to the sensor’s high sensitivity and longevity in harsh and corrosive environments. These attributes offer benefits for numerous applications, including wind power, oil and gas, aerospace, power generation, and structural health monitoring. Various vibration-sensing fiber structures have been demonstrated include fiber Bragg gratings (FBG), Mach-Zehnder interferometers, Fabry–Perot interferometers, long period gratings (LPG), and fiber tapering [3]. The FBG based vibration sensors have a unique wavelength multiplexing capability with high accuracy and sensitivity. However, the fabrication of the FBG or LPG sensors is complicated, expensive, which requires a phase mask and UV laser. The fiber tapering based vibration sensor is fragile, which is not appropriate for some monitoring applications in a harsh environment. Moreover, all of the above fiber structures also have a limited signal-to-noise ratio (SNR), and sensitivity. The single-mode–multimode–single-mode (SMS) fiber structure, which utilizes the multimode interference effect, has been widely investigated for strain, magnetic field, gas/chemical, temperature, and refractive index sensing applications [4]. The SMS fiber structure offers the advantages of low manufacturing costs, high sensitivity, and easy fabrication. The fiber ring cavity laser-based sensor structure has appreciable advantages over other fiber-based structures including higher sensitivity, high optical SNR, and narrow 3 dB bandwidth. Fiber ring lasers combined with interferometer fiber structures have been investigated for measuring strain, temperature, refractive index, and curvature [5,6]. Moreover, the multimode interference fiber structure acts as both the sensing element, and the filter in the ring laser system, simultaneously. In these systems, the sensing performance can be further enhanced by taking advantage of the narrow 3-dB bandwidth (<0.2 nm) of the ring laser output spectrum, and high SNR, [7,8].

Of note, several vibration sensors that operate based on an SMS fiber have been reported. For example, Y. Ran et al. [9] developed a vibration sensor based on the single-mode/no core/single-mode (SNS) structure with vibration frequencies from 100 Hz to 29 kHz and SNR of 40 dB at 500 Hz. J. Guo et al. [10] demonstrated an ultrasonic sensing system based on a tunable fiber laser with phase-shifted FBG (PS-FBG). The proposed tunable ring laser using the PS-FBG sensor has an SNR of 41.7 dB at 200 kHz. Recently, we proposed a fiber ring laser for simultaneous vibration and carbon dioxide sensing, where the vibration sensor is positioned outside of the ring cavity. A measured frequency response from 10 Hz to 50 kHz was realized, and an SNR of 41 dB was achieved at 500 Hz [11]. In 2019, H. Yu et al. [12] demonstrated a liquid-filled photonic crystal fiber-based vibration sensor with a frequency range from 10 Hz to 20 kHz, and an SNR of 33 dB at 600 Hz. In this work, the proposed fiber structure exhibits both high sensitivity, wide frequency response from 10 Hz to 400 kHz; and boasts an SNR of 50 dB at 3 kHz. The fiber optic vibration sensors demonstrated in the literature and proposed method are compared as shown in Table 1.

In this paper, we propose an ultra-sensitive fiber ring cavity laser-based vibration sensor combined with the SMS fiber structure. The proposed fiber structure has the advantage of tuning the ring laser lasing wavelength to the maximum gain region bias point of the SMS transmission spectrum. The laser wavelength sweep is based on a simple fiber loop structure placed into the laser cavity. The output lasing wavelength sweep is realized by varying the displacement on a fiber knot structure using a linear translation stage. The SMS fiber structure is employed in a ring laser cavity, which offers high sensitivity for accurate measurement. This sensor system was deployed in a field-test for pipeline acoustic emissions monitoring, where the SMS sensor was installed on an 18” diameter steel pipeline segment.

## 2. Operating Principle

**Figure 1:**
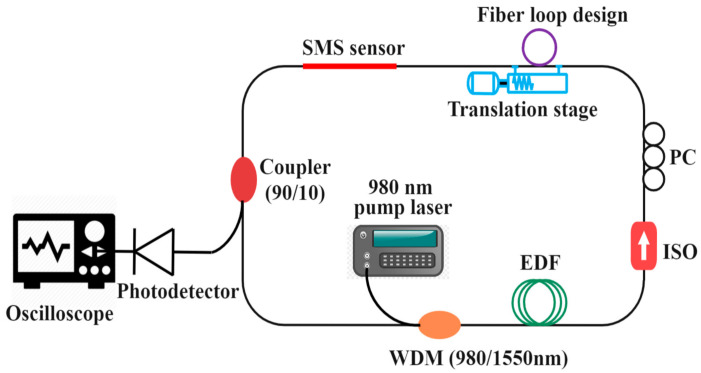
Experimental setup of ultrasonic vibration detection using SMS fiber structure and fiber ring cavity laser.

The SMS fiber structure (shown in Figure 1) acts as a vibration sensor based on the intensity demodulation method. The MMF is fusion spliced between two short sections of SMF and operates based on the multimode interference effect. When the transmitted light fundamental mode, $LP_{01}$ in the SMF, enters the MMF section, the higher-order modes $LP_{0j}$ will be excited. The intensity of the transmitted light spectrum induced by the mode interference is described as [6,14],

(1) $$I(\lambda )=\sum_{i=1}^{N}\eta_{i}^{2}\cdot I_{0}(\lambda )+\sum_{i\neq j=1}^{N}\eta_{i}\cdot \eta_{j}\cdot I_{0}(\lambda )\cdot \cos\left(\frac{2\pi L\Delta n_{eff}}{\lambda }\right)$$

where $I_{0}$ is the fundamental mode intensity in the SMF, λ is the operating peak wavelength, and N is the total number of modes excited within the MMF section. $\eta_{i}$ and $\eta_{j}$ are the coupling efficiency of the fundamental mode $\left(LP_{01}\right)$ and higher-order mode $\left(LP_{0j}\right)$, respectively. $\Delta n_{eff}$ is the effective refractive index difference between the two modes, and L is the MMF length. When the $\left(2\pi L\Delta n_{eff}/\lambda \right)=2m\pi$ condition is satisfied, constructive interference occurs, and the $m^{th}$ order transmitted peak wavelength described as,

(2) $$\lambda_{m}=\frac{L\Delta n_{eff}}{m}$$

where m is an integer. When the strain is applied to the MMF, the phase change will lead to a wavelength shift, $\Delta \lambda_{m}$ given by,

(3) $$\Delta \lambda_{m}=\lambda_{m}\left[\frac{1}{\Delta n_{eff}}\frac{\delta n_{eff}}{\delta \varepsilon }+1\right]$$

where ε=ΔL/L is the strain applied to the MMF, ΔL is the fiber length variation due to the applied strain, and $\delta n_{eff}$ is the photo-elastic induced change in the effective refractive index. If a strain is applied to the MMF section, the fiber length, L will increase and/or decrease. When the MMF is exposed to any vibration signals, the fiber experiences a tensile and compressive strain, as a result, the detected signal intensity increases or decreases [15]. To ensure a high SNR and large amplitude of the measured vibration signal, the ring laser peak wavelength would be lasing within the SMS spectrum maximum gain region [16,17]. By tuning the ring laser lasing wavelength to the optimized maximum gain region bias point of the SMS transmission spectrum, high SNR can be obtained. Therefore, tuning the ring laser output wavelength to an optimized bias point of the SMS spectrum is essential to obtain an enhanced SNR. By creating a fiber loop design in the laser cavity and applying strain to that structure, the lasing wavelength can be tuned. In 2016, Z. Lui et al. [18] demonstrated a fiber strain sensor using a small fiber loop and performed strain sensing with that system. We employed this technique of altering the fiber loop curvature radius to tune the laser output wavelength. The ring laser wavelength here is tuned to a maximum gain medium bias point of the SMS spectrum to get an improved SNR.

## 3. Experimental Setup and Results Discussion

**Figure 2:**
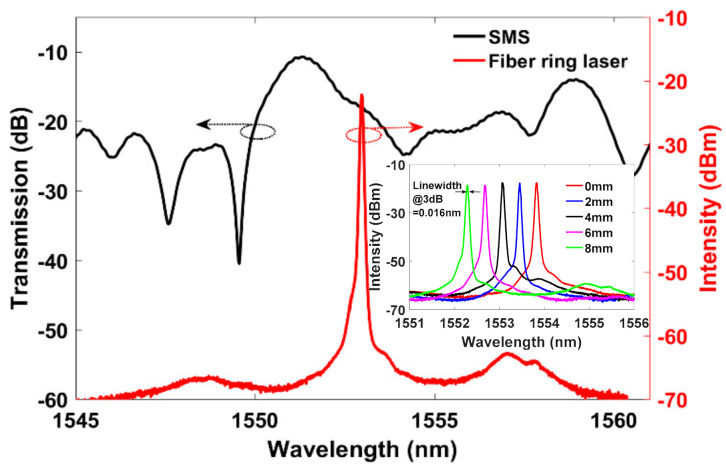
Measured SMS transmission spectrum (black curve) and the laser output spectrum when the SMS sensor inserted into the laser cavity (red curve).

An experimental arrangement for the proposed vibration sensor based on the SMS fiber structure with fiber ring cavity laser is shown in Figure 1. A silica step-index MMF with a length of 8 cm, and a core diameter of 62.5 µm was fusion spliced between two 1 m sections of SMF to realize this SMS structure. A fiber loop is formed in the ring and both ends of the fiber loop are firmly fixed on a linear translation stage as shown in Figure 1. A 980 nm pump laser diode was launched into the ring using a wavelength-division multiplexer (WDM, 980/1550 nm). The ring also contains 10 m of Erbium-doped fiber (EDF). The erbium stimulated emission signal travels through an isolator (ISO) to block unwanted back reflections and ensure uni-directional ring operation. A polarization controller (PC) was used to regulate light polarization in the ring. A 3 dB coupler (90/10) with a high-speed photo-detector was used to out-couple and measure signals. The ring cavity laser output spectrum (at no applied strain), and the measured SMS transmission spectrum were shown in Figure 2. The SMS spectrum was measured using a superluminescent diode (SLD) source with a central wavelength of 1550 nm and a typical 3 dB optical bandwidth of 90 nm. The key advantage of the proposed fiber structure is the simple fabrication of the SMS sensor and accurate lasing wavelength tuning mechanism achieving high SNR. When a filtering structure like the multimode interference-based SMS sensor is employed in the ring cavity laser, the interference spectrum acts as a wavelength selector and allows only one peak to be amplified and produce the single wavelength laser spectrum line. When the SMS sensor experiences external disturbances, such as vibration, the lasing wavelength undergoes intensity modulation. By demodulating and analyzing the laser output signal, the external vibrations can be quantified.

**Figure 3:**
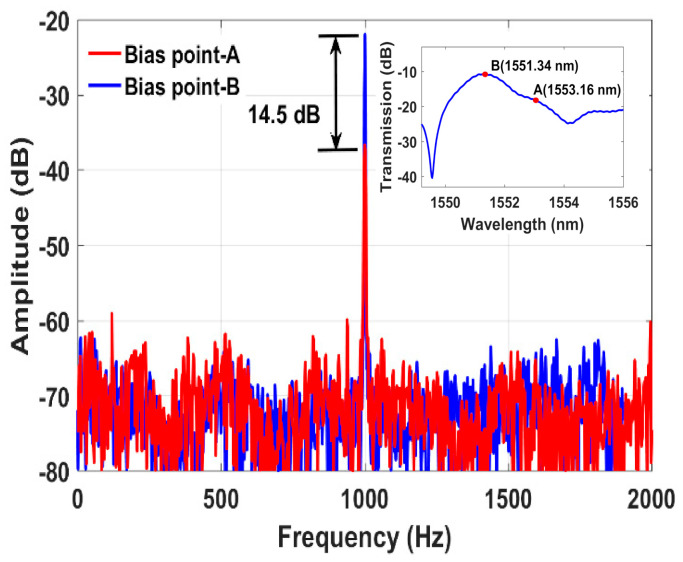
Measured vibration frequency (1 kHz) spectrums at the laser wavelength positioned at two biasing points of the SMS transmission spectrum (inset: SMS sensor transmission spectrum).

The ring laser lasing wavelength consists of a high SNR of 42 dB, and a narrow 3 dB bandwidth of 0.016 nm; which ensures high measurement accuracy. As shown in Figure 2, the laser lasing wavelength (1553.16 nm) does not fall under the resonant peak or maximum gain region of the SMS transmission spectrum, which may be due to some bending of the SMS interferometer [7,19]. To ensure stable laser operation and achieve high measurement SNR, the lasing wavelength should fall within the maximum gain of the SMS attenuation spectrum. By fixing one lead end of the fiber loop and moving the other end using the translation stage, the laser output can be tuned appropriately. Figure 2 inset shows the measured laser output spectrum at various applied displacements. The tuning sensitivity was found to be 214 pm/mm. Hence, the lasing wavelength was tuned from 1553.16 nm (named as bias point-A) to the high gain peak region of 1551.34 nm (named as bias point-B) as shown in Figure 3 inset. The vibration sensing performance was investigated by sweeping the laser lasing wavelength between both biasing points (A:1553.16 nm and B:1551.34 nm) on the SMS transmission spectrum. The SMS sensor was then wrapped around a piezoelectric transducer (PZT) tube actuator as a test platform. The PZT was driven using a function generator to characterize the frequency response and other characteristics of the sensor system. The measured frequency response at a 1 kHz excitation frequency is illustrated in Figure 3. In both bias point conditions, the same set of parameters were used, including a constant laser power of -6 dBm. Operating the lasing wavelength at the maximum gain region (bias point-B) resulted in a 14.5 dB SNR improvement compared to the lasing wavelength operated at bias point-A, as illustrated in Figure 3. Therefore, it is obvious that the maximum SNR can be obtained at the laser lasing wavelength operated at the maximum gain medium of the SMS spectrum. The SNR will become lower when the laser wavelength is tuned away from this value.

**Figure 4:**
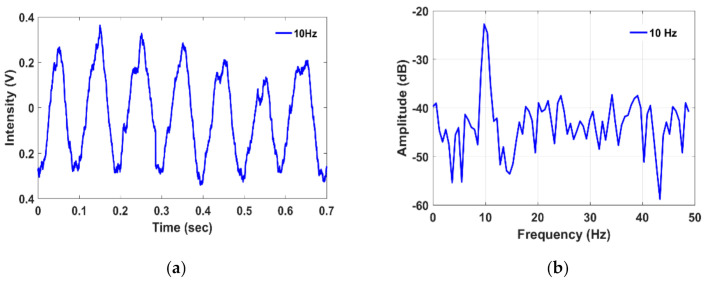
Measured (**a**) time domain and (**b**) frequency domain spectra (obtained using a fast Fourier transform [FFT]) under the 10 Hz sinusoidal vibration frequency.

**Figure 5:**
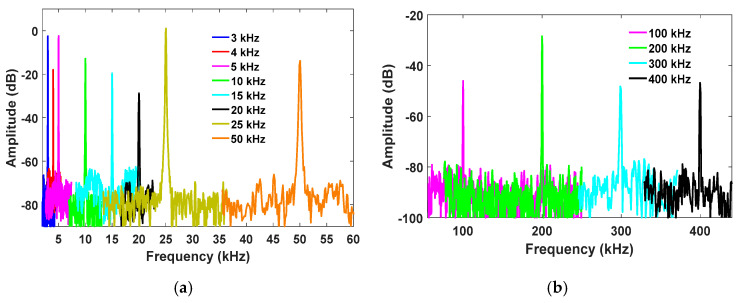
Measured frequency spectra for (**a**) 3 kHz to 50 kHz and (**b**) 100 kHz to 400 kHz.

**Figure 6:**
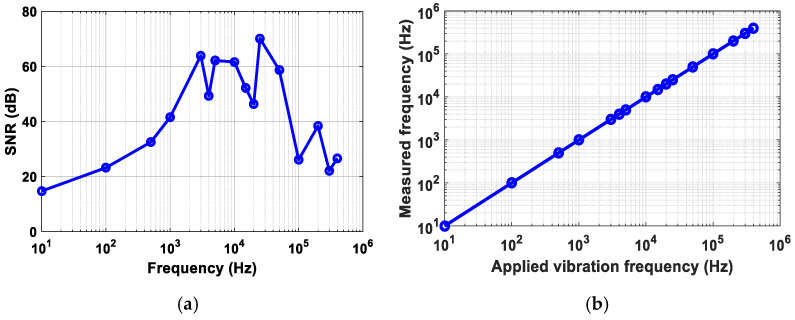
(**a**) Measured SNR as a function of vibration frequency, (**b**) the measured frequencies vs. the applied vibration frequencies from 10 Hz to 400 kHz.

For the remainder of the experiments, the lasing wavelength was fixed at the optimized biasing point (B: 1551.34 nm). Next, the PZT cylinder excitation was set to a 10 Hz sinusoidal signal using a function generator. The measured time domain and frequency domain spectra are shown in Figure 4. Next, the vibration frequencies of 3 kHz, 4 kHz, 5 kHz, 10 kHz, 15 kHz, 20 kHz, 25 kHz, and 50 kHz, were measured as illustrated in Figure 5a. To demonstrate the capability of high-frequency vibration detection, frequencies from 100 kHz to 400 kHz, in increments of 100 kHz, were applied to the PZT. The measured high-frequency spectra are illustrated in Figure 5b. Figure 5 shows the measured amplitude variations over the applied frequencies. The amplitude significantly reduced above the 50 kHz vibration frequency, since the PZT resonant frequency is about 50 kHz and produces reduced amplitude vibrations above that value. Note that the variation of the measured amplitude is frequency dependent. The measured SNR depicted as a function of vibration frequency is illustrated in Figure 6a. The correlation between the measured frequency and the applied vibration frequency is shown in Figure 6b, which illustrates that the proposed sensor can precisely measure the applied frequency.

**Figure 7:**
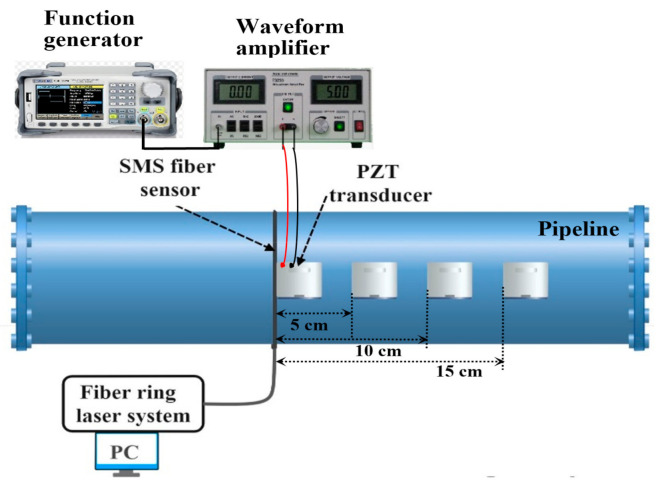
Schematic illustration of the pipeline installed with a proposed vibration sensor system.

**Figure 8:**
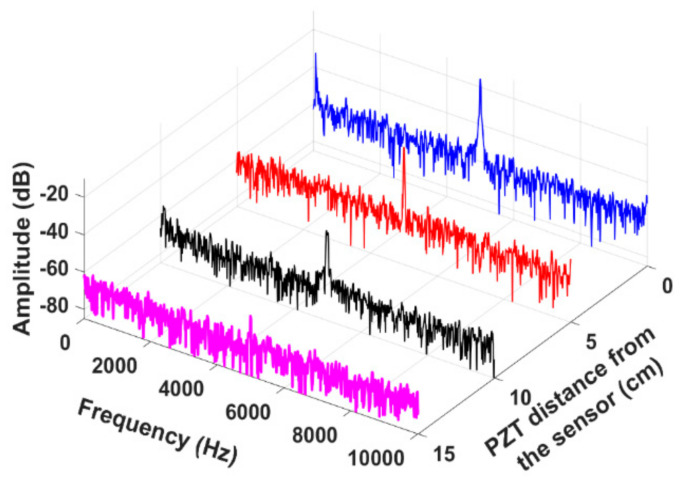
Recorded frequency spectra when pipeline excited by a PZT actuator, positioned at various locations from the sensor.

**Figure 9:**
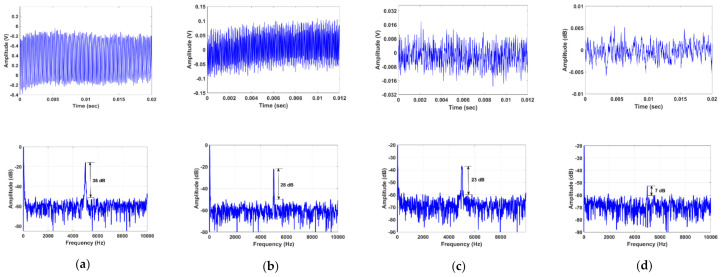
Measured time-domain and frequency-domain spectra of PZT transducer positioned, (**a**) next to the sensor, (**b**) 5 cm, (**c**) 10 cm, and (**d**) 15 cm away from the fiber sensor at a fixed 5 kHz vibrational frequency.

The proposed vibration fiber sensor system was validated in a field application test exploring vibration monitoring on a natural gas transmission pipeline. The pipeline is 18” diameter and made of carbon steel. It was placed 6” above the ground on a set of pipe stands. The SMS fiber sensor was installed by wrapping the sensor element on an empty section of the pipeline as shown in Figure 7. This circumferential sensor configuration enables the monitoring of vibrations due to variations in the tangential (hoop) strain. Hoop strain is the dominant mode of strain under static pressurized loading conditions and is also an excellent indicator of wall defects such as wall thinning and cracks. A PZT actuator (outer diameter:38 mm) was bonded at different locations from the sensor to induce vibrations in the pipeline. The PZT was driven by a function generator and waveform amplifier at a 5 kHz sinusoidal vibration frequency. The PZT continuously vibrates in a controlled way and transfers some of this vibrational energy to the pipeline. The PZT cylinder was placed at locations 5 cm, 10 cm, and 15 cm away from the sensor sequentially. The measured frequency spectra are illustrated in Figure 8. The dominant peak is centered at 5 kHz, which is well matched to the PZT excitation source frequency of 5 kHz. As the pipeline itself attenuates the transmitted vibration along the length, the detected SNR decreases as the PZT is moved away from the sensor. After a 15 cm distance, the fiber sensor no longer picks up the vibrations from the low-power PZT. The recorded time-domain and frequency spectra at various PZT excitation source locations were illustrated in Figure 9. The SNR was measured to be 38 dB, 28 dB, 23 dB, and 7 dB, when the distance was next to the sensor, 5 cm, 10 cm, and 15 cm away respectively. The measured time-domain amplitude and the resultant SNR decrease as would be expected from the carbon-steel material attenuation of these relatively low-frequency acoustic waves over length. These field demonstration results show that the proposed sensor system is capable of dynamic pipeline vibrations monitoring in real field applications [20].

## 4. Conclusions

We have proposed and experimentally demonstrated a high-sensitivity ultrasonic vibration sensing system using an SMS fiber structure, and a fiber ring cavity laser. The proposed fiber structure has the advantage of obtaining an enhanced SNR, which is achieved by the simple tunability of the ring laser wavelength. An improved SNR was obtained when the ring laser wavelength was positioned at the maximum gain region, and SNR degrades when the ring laser wavelength is positioned at a lower-gain region. An SNR enhancement of 14.5 dB was obtained through this tuning procedure. Furthermore, a wide range of detectable frequencies from 10 Hz to 400 kHz was experimentally demonstrated. In addition, the proposed sensor system was successfully field-tested for pipeline dynamic vibration measurement, where the pipeline is excited by a PZT actuator at various locations from the sensor. The proposed technique was shown to be highly capable of dynamic pipeline vibration monitoring for real field monitoring applications.

## Figures and Tables

**Table 1:** Comparison of fiber vibration sensors in literature with proposed fiber structure.

Reference	Sensing Fiber Structure	Frequency Range	SNR
[9]	Single mode-no core-single-mode (SNS)	100 Hz to 29 kHz	40 dB at 500 Hz
[10]	Tunable fiber laser with phase-shifted FBG	200 kHz	41.7 dB at 200 kHz
[11]	Fiber ring laser with external SMS sensor	10 Hz to 50 kHz	41 dB at 500 Hz
[12]	Liquid-filled photonic crystal fiber	10 Hz to 20 kHz	33 dB at 600 Hz
[13]	Tapered SMF	100 Hz to 1 kHz	Not stated
	This work	100 Hz to 400 kHz	50 dB at 3 kHz

## Data Availability

Not applicable.

## Disclaimer

This manuscript was prepared as an account of work sponsored by an agency of the United States Government. Neither the United States Government nor any agency thereof, nor any of their employees, makes any warranty, express or implied, or assumes any legal liability or responsibility for the accuracy, completeness, or usefulness of any information, apparatus, product, or process disclosed, or represents that its use would not infringe privately owned rights. Reference therein to any specific commercial product, process, or service by trade name, trademark, manufacturer, or otherwise does not necessarily constitute or imply its endorsement, recommendation, or favoring by the United States Government or any agency thereof. The views and opinions of authors expressed therein do not necessarily state or reflect those of the United States Government or any agency thereof. The method shown to be capable of practical pipeline monitoring applications, which allows high standards of pipeline safety and integrity monitoring.
